# Supplementary material for: Evaluating the effectiveness and sustainability of a primary healthcare strategy to reduce the prevalence of strongyloidiasis in endemically infected Indigenous communities in Northern Australia
Source: PLoS Negl Trop Dis. 2025 May 30;19(5):e0013136. doi: 10.1371/journal.pntd.0013136 (PMC12148227; doi:10.1371/journal.pntd.0013136)
Supplement: S1 Table — (DOCX) [file pntd.0013136.s001.docx]

###### **S1 Table. *Strongyloides* reports extracted at half-yearly intervals over 4.5 years in four clinics in remote Arnhem Land (July 2012 to December 2016)**. Point prevalence was the number and proportion (% positive) of tested persons (current residents) who were positive at the time of data extraction.

|  | **July 2012 to December 2012** | **July 2012 to June**  **2013** | **July 2012 to December 2013** | **July 2012 to June**  **2014** | **July 2012 to December 2014** | **July 2012 to June**  **2015** | **July 2012 to December 2015** | **July 2012 to June**  **2016** | **July 2012 to December 2016** |
| --- | --- | --- | --- | --- | --- | --- | --- | --- | --- |
| ***Clinic A*** |  |  |  |  |  |  |  |  |  |
| Number of resident adults | 123 | 121 | 128 | 141 | 145 | 160 | 170 | 179 | 195 |
| N (%) tested at least once | 47 (38.2%) | 84 (69.4%) | 100 (78.1%) | 111 (78.7%) | 120 (82.8%) | 132 (82.5%) | 143 (84.1%) | 151 (84.4%) | 164 (84.1%) |
| **N (%) positive on last recorded test;** 95% CI^ | **33 (70.2%);**  55.1 to 82.7 | **55 (65.5%);**  54.3 to 75.5 | **46 (46.0%);**  36.0 to 56.3 | **38 (34.2%);**  25.5 to 43.8 | **31 (25.8%);**  18.3 to 34.6 | **36 (27.3%);**  19.9 to35.7 | **38 (26.6%);**  19.5 to 34.6 | **39 (25.8%);**  19.1 to 33.6 | **29 (17.7%);**  12.2 to 24.4 |
| ***Clinic B*** |  |  |  |  |  |  |  |  |  |
| Number of resident adults | 217 | 183 | 198 | 234 | 254 | 293 | 343 | 409 | 464 |
| N (%) tested at least once | 69 (31.8%) | 101 (55.2%) | 131 (66.2%) | 152 (65.0%) | 168 (66.1%) | 201 (68.6%) | 235 (68.5%) | 277 (67.7%) | 311 (67.0%) |
| **N (%) positive on last recorded test;** 95% CI^ | **45 (65.2%);**  52.8 to 76.3 | **54 (53.5%);**  43.3 to 63.5 | **62 (47.3%);**  38.5 to 56.2 | **51 (33.6%);**  26.1 to 41.7 | **38 (22.6%);**  16.5 to 29.7 | **52 (25.9%);**  20.0 to 32.5 | **59 (25.1%);**  19.7 to 31.2 | **57 (20.6%);**  16.0 to 25.8 | **45 (14.5%);**  10.8 to 18.9 |
| ***Clinic C*** |  |  |  |  |  |  |  |  |  |
| Number of resident adults | 890 | 556 | 552 | 537 | 551 | 556 | 582 | 623 | 774 |
| N (%) tested at least once | 75 (8.4%) | 122 (21.9%) | 140 (25.4%) | 170 (31.7%) | 217 (39.4%) | 255 (45.9%) | 296 (50.9%) | 341 (54.7%) | 410 (53.0%) |
| **N (%) positive on last recorded test;** 95% CI^ | **49 (65.3%);**  53.5 to 76.0 | **80 (65.6%);**  56.4 to 73.9 | **86 (61.4%);**  52.8 to 69.5 | **81 (47.7%);**  40.0 to 55.4 | **68 (31.3%);**  25.2 to 38.0 | **90 (35.3%);**  29.4 to 41.5 | **113 (38.2%);**  32.6 to 44.0 | **113 (33.1%);**  28.2 to 38.4 | **100 (24.4%);**  20.3 to 28.9 |
| ***Clinic D*** † |  |  |  |  |  |  |  |  |  |
| Number of resident adults | 1309 | 1300 | 1225 | 1272 | 1288 | 1305 | 1280 | 1310 | 1375 |
| N (%) tested at least once | 32 (2.4%) | 50 (3.8%) | 78 (6.4%) | 89 (7.0%) | 116 (9.0%) | 263 (20.2%) | 484 (37.8%) | 606 (46.3%) | 724 (52.7%) |
| **N (%) positive on last recorded test;** 95% CI^ | **25 (78.1%);**  60.0 to 90.7 | **39 (78.0%);**  64.0 to 88.5 | **55 (70.5%);**  59.1 to 80.3 | **56 (62.9%);**  52.0 to 72.9 | **56 (48.3%);**  38.9 to 57.7 | **104 (39.5%);**  33.6 to 45.7 | **167 (34.5%);**  30.3 to 38.9 | **179 (29.5%);**  25.9 to 33.4 | **162 (22.4%);**  19.4 to 25.6 |

^95%CI = 95% exact binomial confidence intervals. †Clinic D joined the study in January 2015: prior to this date, serological tests for strongyloidiasis were only requested if the clinician suspected an infection.
