# Supplementary material for: Evaluating the effectiveness and sustainability of a primary healthcare strategy to reduce the prevalence of strongyloidiasis in endemically infected Indigenous communities in Northern Australia
Source: PLoS Negl Trop Dis. 2025 May 30;19(5):e0013136. doi: 10.1371/journal.pntd.0013136 (PMC12148227; doi:10.1371/journal.pntd.0013136)
Supplement: S2 Table — 2012–2016. (DOCX) [file pntd.0013136.s002.docx]

###### **S2 Table. Analysis for each clinic and overall, for 680 persons with at least one positive test. 2012–2016**

|  | **Clinic A** | **Clinic B** | **Clinic C** | **Clinic D** | **Overall Total** |
| --- | --- | --- | --- | --- | --- |
| Number of persons who were **positive at least once** | **88** | **126** | **214** | **252** | **680** |
| Number of persons who were tested once and found to be positive, with no follow-up test | 17 | 28 | 75 | 133 | 253 |
| N (%) Persons who **were eligible for assessment of follow-up**  (680 - 253 = 427) | **71**  (80.7% of 88) | **98**  (77.8% of 126) | **139**  (65% of 214) | **119**  (47.2% of 252) | **427**  (62.8% of 680) |
| Number of persons who were positive at end date of study, excluding those with only one test | 15 | 20 | 39 | 33 | 107 |
| N (%) Persons who were positive at least once and **negative at end of study** as proportion of those with more than one test.  (427-107=320) | **56**  (78.9% of 71) | **78**  (79.6% of 98) | **100**  (71.9% of 139) | **86**  (72.2% of 119) | **320**  (74.9% of 427) |
