# Supplementary material for: Evaluating the effectiveness and sustainability of a primary healthcare strategy to reduce the prevalence of strongyloidiasis in endemically infected Indigenous communities in Northern Australia
Source: PLoS Negl Trop Dis. 2025 May 30;19(5):e0013136. doi: 10.1371/journal.pntd.0013136 (PMC12148227; doi:10.1371/journal.pntd.0013136)
Supplement: S3 Table — 2012–2016. (DOCX) [file pntd.0013136.s003.docx]

###### **S3_Table. Breakdown for each clinic for reinfection and number positive on last test, 2012–2016**

|  | **Clinic A** | **Clinic B** | **Clinic C** | **Clinic D** | **Total** |
| --- | --- | --- | --- | --- | --- |
| Number of persons who tested positive with more than one test | **71** | **98** | **139** | **119** | **427** |
| N (%) Persons with reinfection during follow-up time | 11  (15.4% of 71) | 7  (7.1% of 98) | 12  (8.6% of 139) | 3  (2.5% of 119) | 33  (7.7% of 427) |
| N (%) Persons who were re-infected and remained positive at end of 2016 | 5  (7% of 71) | 4  (4% of 98) | 7  (5% of 139) | 1  (0.8% of 119) | 17  (3.9% of 427) |

###### 
