# Supplementary material for: Evaluating the effectiveness and sustainability of a primary healthcare strategy to reduce the prevalence of strongyloidiasis in endemically infected Indigenous communities in Northern Australia
Source: PLoS Negl Trop Dis. 2025 May 30;19(5):e0013136. doi: 10.1371/journal.pntd.0013136 (PMC12148227; doi:10.1371/journal.pntd.0013136)
Supplement: S4 Table — (DOCX) [file pntd.0013136.s004.docx]

###### **S4 Table. Final Evaluation 2012–2020.** Only current residents on 31 December 2020 were included in this table.

|  | **Clinic A** | **Clinic B** | **Clinic C** | **Clinic D** | **Total** |
| --- | --- | --- | --- | --- | --- |
| Number of current resident adults | 217 | 389 | 727 | 1510 | 2843 |
| N (%) tested at least once between 2012 and 2020; 95% CI^ | 209 (96.3%);  92.9 to 98.4 | 310 (79.7%);  75.3 to 83.6 | 625 (86%);  83.2 to 88.4 | 1246 (82.5%);  80.5 to 84.4 | 2390 (84.1%);  82.7 to 85.4 |
| **N (%) positive for *Strongyloides* at least once between 2012 and 2020;** 95% CI^ | **117 (56%);**  49.0 to 62.8 | **132 (42.6%);**  37.0 to 48.3 | **330 (52.8%);**  48.8 to 56.8 | **477 (38.3%);**  35.6 to 41.0 | **1056 (44.2%);**  42.2 to 46.2 |
| **N (%) positive for *Strongyloides* on last recorded test;** 95% CI^ | **9 (4.3%);**  2.0 to 8.0 | **19 (6.1%);**  3.7 to 9.4 | **80 (12.8%);**  10.3 to 15.7 | **124 (10%);**  8.4 to 11.7 | **232 (9.7%);**  8.5 to 11.0 |

^95%CI = 95% exact binomial confidence intervals
