# Supplementary material for: Evaluating the effectiveness and sustainability of a primary healthcare strategy to reduce the prevalence of strongyloidiasis in endemically infected Indigenous communities in Northern Australia
Source: PLoS Negl Trop Dis. 2025 May 30;19(5):e0013136. doi: 10.1371/journal.pntd.0013136 (PMC12148227; doi:10.1371/journal.pntd.0013136)
Supplement: Attachment — Submitted filename: Response to Reviewers PNTD-D-24-01494 R1.docx [file pntd.0013136.s006.docx]

# Response to Reviewers PNTD-D-24-01494

| **Reviewer** | **Response** |
| --- | --- |
| **Methods:** |  |
| Reviewer #1: *All questions and criteria are met in the manuscript.* | Thank you. |
| Reviewer #2: *It would be good to provide more information about the ELISA testing.*  *How were the results standardized over time?*  *What was the turnaround time before results were returned to the clinic?* | The same commercial IVD ELISA was used for every test from July 2012.  See Diagnostic test track changes: lines 191and 192.  “This aligned with the introduction by Western Diagnostic Pathology (Perth, Western Australia) of the IVD enzyme-linked immunosorbent assay test (ELISA) (DRG Instruments GmbH, Marburg, Germany) which was used for all testing in this study (41)”  The laboratory used was Western Pathology which is accredited by NATA (National Association of Testing Authorities), the entity responsible to ensure laboratories meet national standards.  Usually about one week. Pathology is sent from remote clinics by plane to Darwin usually within 24 hours, then forwarded to Western Diagnostic Laboratory in Perth, Western Australia. Results are sent electronically into Electronic Health Record System (Communicare), checked by the doctor, then a recall for treatment entered into the electronic health record, if positive result. |
| **Results:** |  |
| *Reviewer #1:* *All questions and criteria are met in the manuscript.* | Thank you. |
| *Reviewer #2: Are the data sufficient to look at incidence?*  *What do the communities think about the project?* | The study was not designed to measure incidence, but to continue testing to identify positive cases that require treatment, whether due to reinfection, new infection, or inadequate treatment.  The data only gives an indication of incidence based on new infections and reinfections for those who had more than one test during 2012-2016 i.e. 427 persons. 13 of 427 (3%) were neg to positive and categorised as new infections, and 33 of 427 (7.7%) pos-neg-pos considered reinfected. Between 2012 and 2016, 75% of those with a follow up result declined to negative. The report from 2012 to 2020 shows that this improved to 85% (Fig 5).  As stated in the introduction, “Aboriginal leaders requested action, stating they did not want to suffer from a preventable disease that is rare in mainstream Australia”. Regular reports to the Yolngu Miwatj Board received endorsement and positive feedback when results were shared. |
| **Conclusions:** |  |
| Reviewer#1: *All questions and criteria are met in the manuscript.* | Thank you. |
| Reviewer#2: *What has been done to scale up the intervention to other communities?* | We have scaled up this intervention in all seven health services which now come under Miwatj Health Aboriginal Corporation. It is embedded in the system.  We plan to advocate for other health services in endemic communities to adopt this system, using this PLOS NTD paper for evidence.  Also note Generalisability section (Lines 436-439). |
| *Why doesn’t Australia procure WHO prequalified generic ivermectin?* | That is a very good idea. The Pharmaceutical Benefits Scheme (PBS) in Canberra make those decisions. Currently the PBS is $26 for onchocerciasis, and $40 for strongyloidiasis from Merck (during this study). In our setting, the patient does not pay for medications due to our very remote location. Ivermectin is dispensed from the remote clinics.  We think PBS will be interested to access the WHO prequalified generic ivermectin. There will also be interest in an Albendazole + Ivermectin combination, as it is likely to have a synergistic benefit in control programs. The current deworming program for children is Albendazole and combining it with ivermectin would make it more effective against strongyloidiasis, and address the gap of children not receiving ivermectin in this program.  See Limitations section lines 527-530. |
| **Summary and General Comments** |  |
| Reviewer #1: *The manuscript “Evaluating the effectiveness and sustainability of a primary healthcare strategy to reduce the prevalence of strongyloidiasis in endemically infected Indigenous communities in northern Australia describes the use of serology for Strongyloides combined with treatment as a strategy for controlling strongyloidiasis in an endemic area with an Indigenous population. The United Nations 2030 Agenda for Sustainable Development aims to end epidemics of AIDS, tuberculosis, malaria, and tropical diseases, such as soil-transmitted helminthiasis. However, within the context of soil-transmitted helminthiasis, efforts are primarily focused on ascariasis, trichuriasis and hookworm , with little attention given to the control of strongyloidiasis. In the manuscript from northern Australia, the authors present an experience report that shows a reduction in disease rates, which seems to be a promising strategy, though it requires further follow-up to be validated.*  *The manuscript is written in a simple and straightforward manner, and although the data presented in this paper are linked to a previous publication, it is still understandable.*  *Below are some considerations regarding the manuscript*. | Thank you very much for your positive feedback and considerations to increase understanding of this strategy. |
| *Q1. The data in Table 1 and S1 table need to be reviewed. This also impacts the specific values described in the sections “Final evaluation 2012 -2020” and “Analysis of data from the implementation phase 2012-2016.”* | The outcome measures were in three phases, as described in methods study design. The differences in the data shown in S1 Table (phase 1) and Table 1 (phase 2) are due to the dynamic, real-world population studied, and the progressive collection of data. Figure 3 and S1 table reflect the results of only current residents at the time of half-yearly data extraction from the basic EHR reports. Figure 3 title added “for current residents at the time of data extraction.”  The comprehensive analysis 2012-2016 in Table 1 title was clarified to for all persons tested.  The analysis of data section Lines 303-307 now clarifies “This statistical analysis included all persons tested at least once, including 77 persons who were no longer residents in communities at the end of 2016.”  As coverage increased, positive results decreased, but that was insufficient to measure if the change in prevalence was due to the intervention. The period prevalence was not measured during the implementation period and was only measured when all results were put into the statistics package (phase 2). Comparing ever-positive to positive on the last test was important.  In 2020, the EHR report was then improved to include ever-positive, so that increase in coverage and change in prevalence (ever-positive compared to positive on last test) were measured directly. This improved EHR report (Table S4) extracted data only on current adult residents in 2020 and was then used to evaluate the sustainability four years after the formal intervention study.  To improve clarity, text in results sections (during the implementation phase and analysis of all data from the implementation phase from 2012-2016) have been modified, as above. The final evaluation section has also had minor modifications to bring clarity to three phases. The data for the three phases have been reviewed and remain unchanged.  We hope the detailed methods will assist others with implementing, monitoring, evaluating and surveillance of their own community *Strongyloides* control programs. |
| *Q2. Were the 13 individuals who had a negative diagnosis in the previous serology, but tested positive in a new test, treated?* | Yes, the protocol is for any positive result, the patient is recalled for treatment.  See Reinfection and new infection section where this is now stated clearly in Lines 327-328 “The ongoing protocol is for any positive result, the person is recalled for treatment.”  Thank you. |
| *Q3. Were the 17 individuals who remained serologically positive after treatment retreated?*  .  *What was the protocol for cases where individuals remained positive throughout the periodic testing?* | Yes, the protocol is for any positive result, the patient is recalled for treatment. There is at least a six-month delay between treatment and opportunistic follow-up serology result, thus the positives who were treated would remain positive on the latest report until they had another serology test.  As above, the protocol is for any positive result, the patient is recalled for treatment.  i.e. Patients whose OD had declined, but not yet negative (OD below 0.2) at any visit, were re-treated with the same dose. |
| *Q4. In endemic areas, the main hypothesis for persistent infection is reinfection, but we cannot exclude the possibility of parasitic resistance, especially considering the limited therapeutic options available for the treatment of these infections. Therefore, it is important to discuss this issue in the manuscript, and to acknowledge the inability to distinguish between reinfection and resistance as a limitation of the study.* | We agree with this point and have now acknowledged it in the limitation section Lines 535-540. “This population health-systems intervention did not have the capacity to investigate de-identified individuals. The smaller number of remaining positive individuals could be further researched for reasons such as adherence to treatment, inadequate treatment, resistance to treatment or investigating sources of environmental reinfection. Those individuals not responding to multiple doses of ivermectin may be suited to second-line future treatment options such as ivermectin/albendazole combination or moxidectin”. |
| Q5. The authors state *“Persons who stayed positive were significantly younger (median age 31) compared with persons who became negative (median age 39)”.*  *“Reinfected persons were on average older (median age 42), compared with no reinfection (median age 35)* ”. *Is there any hypothesis to explain this finding?* | This is an observation from the statistical analysis, but differences in age were beyond the scope of this project.  As younger persons had less contact with the health service, and follow-up serology was opportunistic, thus the positive result would have remained until the follow up test which may have been much later. The delay in the largest clinic joining the intervention may contribute to this observation, due to the shorter time period for follow-up (Line 285-287). Older persons are more likely to attend the clinic with other chronic diseases, so reinfections were more readily detected over a longer time period. |
| *Q6. Were health education strategies employed during the study? Typically, education activities are crucial for reducing prevalence in endemic areas where selective treatment is carried out*. | Yes, they were. Community education was an ongoing effort both before and during the project. (Line 174-176).  “Health education strategies and resources were developed and used prior to the study as part of the engagement and co-design process, and also throughout the study to educate both clinicians and patients.”  Health resources for the community had previously been developed, such as a flip chart story and local radio messages in local language. Community awareness was relatively high, but a high turnover and low awareness of Australian health professionals made the development of educational resources for medical professionals the priority for this project. In addition, the misperception of an indefinite environmental reservoir of infection was identified as a barrier and addressed by CDC in 2015, and the background to understanding the unique lifecycle of Ss was published in 2018. (Reference #17) |
| *Reviewer 2: In this manuscript, Page and colleagues report on the sustainability of a Strongyloides control program implemented through the routine health care system in Indigenous communities in Australia. The results show that this strategy results in a dramatic reduction in disease prevalence over time*. | Thank you very much for your constructive comments and positive review. |
